# Methodology to Reduce Distortion Using a Hybrid Thermal Welding Process

**DOI:** 10.3390/ma11091649

**Published:** 2018-09-07

**Authors:** Javier Souto, Enrique Ares, Paulino Alegre, Jorge Cerqueiro

**Affiliations:** 1 Calculus and Simulation Unit at AIMEN Technology Center, C/Relva 27A Torneiros, 36410 Porriño-Pontevedra, Spain; 2 Escuela de Ingeniería Industrial, Campus Universitario, University of Vigo, 36310 Vigo-Pontevedra, Spain; enrares@uvigo.es (E.A.); alegre@uvigo.es (P.A.); jcerquei@uvigo.es (J.C.)

**Keywords:** welding distortion, FEM, LSND (low stress no distortion), htTTT, model validation

## Abstract

Welding is a thermal process which results in high strain and stress values in the material and leads to its change of phase. This might cause significant distortions in the welded structure, which often becomes a relevant design and manufacturing issue. This study deals with a line-heating-based forming process that is applied at the moment of the welding operation, with the final objective of minimizing distortion. A FEM (finite element method) based on a thermo-elastoplastic approach is used here. The computational method is first calibrated in three stages: heatline forming calibration, flame heat source calibration, and the weld process. The final model presented in this work simulates a hybrid process called htTTT (high-temperature thermal transient tensioning) which was optimized over large T welds to minimize the final distortion of the components.

## 1. Introduction

Welding is a joining process which generates changes in the material state at high temperatures. Welding technology is widely used in many different industries, such as shipbuilding or the nuclear or automotive industries. Welding has interesting properties with respect to other joining technologies: its continuity in the final assembly, speed and versatility in the design and fabrication stages, etc. However, this technology in its traditional form has disadvantages because the high temperature involved generates thermal expansion, shrinkage, and microstructural transformations. For that reason, the welded joints in structures need constant supervision by highly qualified workers.

The prediction and control of distortion is particularly important for the design and manufacturing of stiffened welded structures [1]. The extensive use of fusion-welding processes in the manufacturing of this kind of structures generates high temperature in the parts, resulting in high distortion in the final assemblies. This undesirable effect leads to direct costs due to the reworking that is needed to assure the compliance of the final product. Thus, numerical simulation becomes an essential tool for the forecasting and control of distortion in order to avoid high reprocessing costs in the fabrication stage.

The distortion phenomenon has been studied to develop new ways of minimizing its effects. A reduction of the distortion can be achieved through different strategies based on treatments applied either previous to, throughout, or after the weld process. These techniques can be mechanical [2], thermal [3], electromagnetic [4] or use other welding processes [5,6], and can be based on minimizing net stress in the assembly or inducing additional stresses after the welding process, such as in straightening.

Avoiding the distortion phenomenon by using predictive methods is an actual challenge that is addressed in this work. The thermal transient tensioning (TTT) process was studied for the case of a typical shipbuilding stiffener. Parametric analysis for different torch positions and heat power densities was performed. The result will point to a new constructive technique which aims to reduce cost and construction time.

The transient thermal tensioning process was patented by Dull, R.M. [7] (Edison Welding Institute, Columbus, OH, USA). This process is one of the more promising techniques used to mitigate the distortion produced by welding processes. In this method, different heat sources are located in different areas over the plates. These sources are transient, thus implying the presence of moving and localized heat sources, which produce both temperature gradients and thermal stresses. The substantial idea of TTT is to control the stresses in the assembly during the welding by combining the auxiliary heat sources with the welding process heat sources.

## 2. Method Details

In this work, a modified TTT procedure was developed and applied to a large T-joint. High-temperature TTT (htTTT) was applied over a typical stiffener used in the shipbuilding industry, but the conclusions are relevant to other industries where these kinds of assemblies are used.

**Figure 1:**
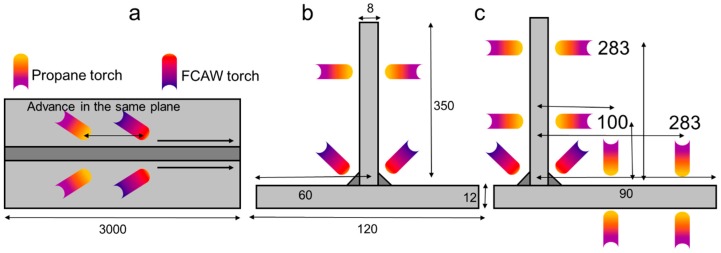
Schematic of the manufacturing process with the size of the T-joint. (dimensions in mm). (**a**) Distance between process torches; (**b**) P1 geometry; (**c**) P2 geometry.

The T-joint (P1) and the asymmetrical T-joint (P2) are assemblies of two plates, each 3 m long (Figure 1). The difference between both assemblies lies in the position of the web over the flange. Long T-joint stiffeners are sometimes welded in several bead depositions using specific sequences [8]. Those sequences can minimize the total distortion, but the process is slower and complex, and therefore other strategies to avoid the distortions were studied.

The reference assemblies (P1 and P2) are welded by using double-sided symmetric flux cored arc welding (FCAW) welding. The weld was performed in a single pass from start to end, and therefore the total time for the weld process was short. The TTT process was applied to P1 and P2 (Figure 1); the use of the transient heat-forming torches together with the welding process is specific to the TTT process. The technique implemented in this case involved using two symmetrical propane torches over the FCAW torches aimed towards the web of the structure, as shown in Figure 1. Two torches were used to avoid asymmetrical thermal loads, so that, at the end of the process, no transversal deflection was observed.

The TTT developed for this case was a stressing method; it operates as with heatline forming, using a high thermal power to introduce strains/stresses into the plates. This operation is different from the low stress no distortion method [9], where the minimization of the final residual stresses implies lower distortions.

The case described here shows a high-temperature TTT process, where the residual compressive stresses work against the weld distortion. The reduction of distortion was controlled by acting on the flame intensity and the position of the flame torches. The distortion produced by both the weld and the flame heating will depend on the geometry and the thermal power of the heat sources; therefore, to obtain the optimal final non-distortion configuration, it will be necessary to calibrate the position and the power of both sources.

The clamping strategy for the TTT process studied in this research involves minimum clamping, implying that the assembly rests on a surface without any geometrical restriction. The flange and the web of the T assembly must be correctly positioned and joined by short welds; this will prevent separation or misalignment of the plates.

The distance of the torches to the plates and the power of each single torch should be carefully controlled to ensure that the power acting on the plates is symmetrical; also, the heat power applied on the plates must be controlled because a low power of the flame torches will produce low distortion correction, while a high power could yield higher final distortions. This effect is investigated in detail in the following sections.

The advantage of TTT comes from being an in-process method; therefore, from the viewpoint of manufacturing efficiency and cost, this process is more desirable than other alternative processes such as straightening. The process studied in this research was applied together with the weld at the same time and same speed as the weld torch; therefore, the total time of manufacturing was the one-pass welding time.

## 3. Methodology

The welding/heat forming simulation problem has a complex nature. The process is highly nonlinear and it couples together thermal, mechanical and metallurgical fields: thermal loads induce changes in the mechanical fields, while high temperatures and high cooling rates can lead to phase change and metallurgical transformations which make the material properties dependent on the temperature and metallurgical phase proportion. The geometry was meshed using 132.642 hexahedron elements. No rigid clamping was used; instead, the T-joint was simply supported. The constant convection film coefficient (5 W/m^2^) was defined for the exterior surfaces of the assembly. The model was built using the ESI.Syweld software (2014, ESI Group, Paris, France).

However, it is not possible to simulate all of the physics involved in the welding process in a realistic way, as detailed in the study by Lindgren [10]. At present, the FEM simulations for welding often use a Lagrangian mesh [1,2,3,4,5,6,7,8,11]. In this work, the heat source was modeled with the Goldak [12] approximation (Equation (1)) for the weld heat input, and the propane torch source was modeled by 3D conical approximation. The thermo-metallurgical-elastoplastic model approximation (2) has showed great accuracy for weld simulation as well as line forming [13], but in the case of big assemblies, the computation cost increases significantly.

(1) $$P=\int Q(x,y,z,t)=Q_{f}\cdot e^{-\frac{{\left[x+v\left(\tau -t\right)\right]}^{2}}{a_{f}^{2}}}\cdot e^{-\frac{y^{2}}{b^{2}}}\cdot e^{-\frac{z^{2}}{c^{2}}}+Q_{r}\cdot e^{-\frac{{\left[x+v\left(\tau -t\right)\right]}^{2}}{a_{r}^{2}}}\cdot e^{-\frac{y^{2}}{b^{2}}}\cdot e^{-\frac{z^{2}}{c^{2}}}\;$$

(2) $$\overset{•}{\varepsilon =}\overset{•e}{\varepsilon }+\overset{•p}{\varepsilon }+\overset{•tp}{\varepsilon }+\overset{•th}{\varepsilon }\;$$

In the equations above, Q is the power of the source, $Q_{f}$ and $Q_{r}$ are the energy in the front and rear zones of the source, respectively, a, b and c are the dimensions of the elliptical shape of the source, r is the radius of the Gaussian source over the plate, v is the speed of the source and x, y and z are the position coordinates of the source over the plate. The weld beads were added during the process using the rebirth elements method [14]. The present study is performed taking the strains in Equation (2) into account: the strains due to the elastic and plastic domain ($\overset{•e}{\varepsilon },\overset{•p}{\varepsilon }$), thermal strains ($\overset{•tp}{\varepsilon }$) and strains produced by the phase transformations ($\overset{•th}{\varepsilon }$). The welding process can be a cyclic process (multi-pass welding) and therefore the plasticity was calculated using a linear kinematic hardening approximation for the elastic-plastic constitutive model [15]. The material (DH-36) was characterized at the AIMEN Technology Centre facilities (Porriño-Pontevedra, Spain). Its properties—specific heat, thermal conductivity, young modulus, elastic limit and stress–strain curves—were temperature-dependent (0–800 °C), and values higher than 800 °C were extrapolated from those. These values were completed using other ones from references [16].

The htTTT methodology was simulated using a highly non-linear model with large-displacement and large-strain behaviors. Also, the materials were modeled with non-linear properties, and therefore the thermo-metallurgical-mechanical model used to compute the weld process was executed with the updated Lagrangian formulation and Newton–Raphson method [13].

**Figure 2:**
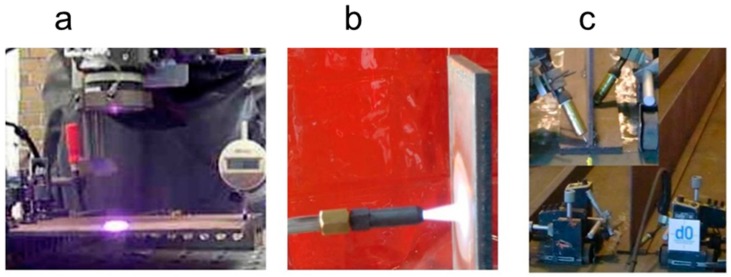
Experimental procedures, (**a**) heatline laser forming process; (**b**) propane heating process; (**c**) flux cored arc welding (FCAW) welding process.

First, the three initial experimental procedures were carried out (Figure 2) and characterized and validated by FEM, as described below. Small laboratory tests were used in the first and second stage. For the final stage, some representative structures in the shipbuilding industry were welded. Naval grade steel (DH-36) was used in every experiment. Shipbuilding process parameters (current, voltage and torch speed) were used depending on the thickness, weld process and materials. The experiments were performed at the AIMEN Technology Centre facilities. The results from the experimental tests were used to validate the numerical models [17].

1. The heatline forming process using a heatline laser diode was applied over small plates (300 × 200 mm) with two thickness values (6 and 8 mm) under different power density configurations. The assembly chosen was a cantilever-like structure. The laser scanning was performed in the middle line of the plate;
2. The propane gas static heating process characterization was performed, using a heating propane torch over a vertical plate. The heating of the heatprint and the cooling the plate were analyzed;
3. Weld process characterization over a large T-joint using a FCAW (flux cored arc welding) process. The tests were performed with continuum and double-sided welds. Every sample used was three meters long. The thickness values chosen were 8 mm for the web and 12 mm for the flange.

Every experiment was recorded with a thermal camera to analyze the evolution of the temperature field; also, the dimensions of every sample were measured before and after the thermal process, as was the flatness of each component. The plates were carefully positioned and measured to ensure the correct initial state, because any misalignment or deviation would generate inconsistencies in the distortion. In this work, the maximum vertical deflection was chosen as a reference to verify the accuracy of the FEM model chosen. The experimental results were used to validate the FEM model and to generate the htTTT numerical model [17].

**Figure 3:**
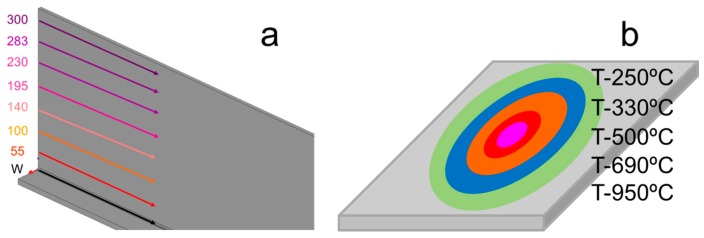
(**a**) Vertical parametrization in mm; (**b**) Temperature objective parametrization.

The numerical model was based on the mathematical description from [14,15,16,17,18,19,20,21]. The model of the htTTT process was developed incorporating the flame torches to the weld process. In this work, the four-heat-sources approach is shown with two welding torches plus two propane torches. For the htTTT comparison, the welding process used was the same for every case, but the heatline torches’ powers and positions were varied in the study. Seven different positions–heights (mm) for the flame torches over the web of the assembly (Figure 3) were studied.

Five different power density values (Figure 3) were applied in this study, while only the net power was changed in the definition of the source model. It is not possible to measure the net power experimentally because the experimental propane setup is carried out by mixing the volume and the pressure of oxygen and propane, so the torches were identified by their corresponding maximum temperature produced on the plate (Figure 3). The maximum temperature is the resulting combined effect of the power source and the velocity of the torch and the thickness of plate.

The power, velocity, and therefore temperature was almost constant throughout the whole process. The trajectories of the two pairs of torches—welding and TTT—were parallel and their displacements synchronized, and all four torches therefore advanced with the same direction and at the same speed.

## 4. Results

**Figure 4:**
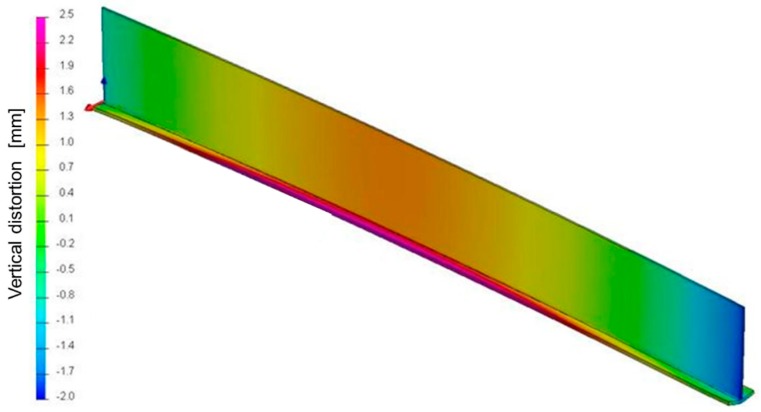
Vertical deflection of P1, the reference T-joint. Scale of vertical distortion (mm).

A de-coupled thermo-mechanical method was used to predict the TTT behavior and for the calculations for the different torch positions over the web. Figure 4 shows the maximum vertical deflection for the parametrization of the TTT torch position and heat power.

**Figure 5:**
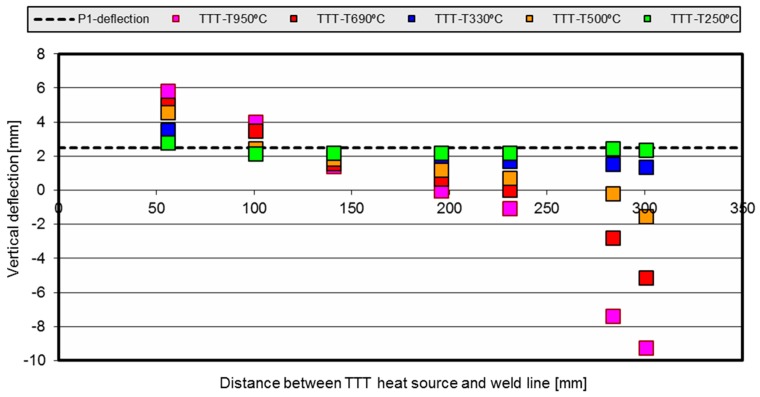
Vertical deflection comparison against torch temperature and torch position parametrization.

The Figure 5 shows the numerical simulation results of the htTTT compared against the experimental results of the welded P1. The black dashed line represents the distortion previously calculated and validated against the experimental results (corresponding to the P1 welding process alone). The colored squares (datapoints) show the distortion obtained by the numerical simulation model results for each htTTT parameterization. In the graph, the *y*-axis indicates the representative distortion value while the *x*-axis shows the position of the TTT torches over the length of the web.

For instance, the red dots indicate that both the HL-55 and HL-100 cases show a higher final distortion value when the TTT torch is too close to the joint. When the TTT torch is 140 mm up the weld joint, an improvement in the final distortion is noticed. For HL-230, the distortion after the process is almost zero, while above 230 mm over the joint base, the final distortion obtained is negative, meaning that it is produced in the direction opposite to the natural one caused by the welding process.

Similar trends were observed for the blue, orange, red and pink curves. Higher distortion values were obtained with the TTT torches positioned closer to the weld torches, while those values decreased as the position of TTT torches was further away from the weld line. The behavior of the distortion is amplified for higher temperature values, so the slopes of the curves are higher. For the lower TTT temperature studied here—green dots—the performance is small, with the points laying very close to the black line. Surprisingly, the optimal distortion behavior for the green points happened for the HL-100 case due to the thermal coupling because of the reduction in the thermal gradients around the weld zone.

It is possible to extract from the dots’ tendencies, shown in Figure 5, the behavior of the structure for each case, and to calculate the optimal torch position to obtain null distortion: 198 mm for TTT-950 °C, 231 mm for TTT-690 °C and 283 mm for TTT-500 °C. The cases with source power TTT-330 °C and TTT-250 °C do not reach the zero distortion value for the P1 geometry.

It can be seen that the TTT action may reduce or increase the final distortion. Therefore, there should be an inflexion point between both behaviors, and that point is called the ‘neutral zone’. As shown in these figures, the position of that neutral zone depends on the heat power and the geometry of the plates, and the dependency is strongly non-linear.

**Figure 6:**
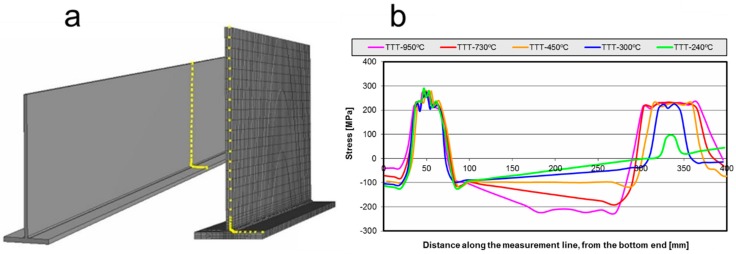
(**a**) Measurement points for the residual stresses; (**b**) Residual stresses over the measurement line for thermal transient tensioning (TTT)-HL283.

The residual stresses were extracted from the FEM postprocessing. The stresses were measured on the surface of the plates located in the middle of the assembly (Figure 6). The black line shows a reference solution representing the action of the welding process alone. The stresses over the bead are similar in every case, with the major differences appearing in the place upon which the propane torch acts. Increasing the source heat power results in higher stress values and a wider affected zone. Moreover, it produces higher compressive stresses along the plate. This is a clear difference that can be seen between the conventional welding and the TTT process. The welding and TTT processes together induce high tensile stress in the position where the propane torches are working. Thus, two main zones with peak stress values can be seen to appear for the welding-plus-TTT process.

The green line shows a small stress peak at the propane torch position due to the low-power source applied in that case. It can be seen that, except for the torch position, the longitudinal stress curve is parallel to the welding stress curve, the final distortion being the same in both cases. With the high-power source, lower peak stress values are reached for the bead position, but that difference is almost negligible, and so the distortion reduction is performed by the introduction of the compressive stresses.

**Figure 7:**
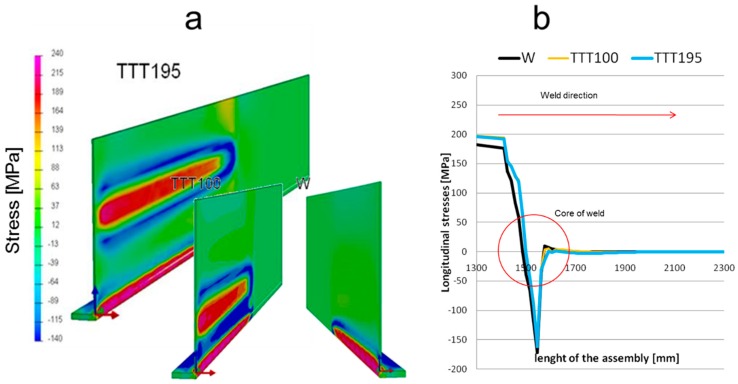
(**a**) Stresses distribution during the high-temperature TTT (htTTT) process (MPa); (**b**) stress distribution during the process.

The reduction in the final distortion was caused by the compressive stresses from the auxiliary thermal sources. Comparing the results along the weld line (Figure 7b), the longitudinal stress shows the same values and trends. Therefore, the TTT applied does not affect directly the action of the weld pass.

**Figure 8:**
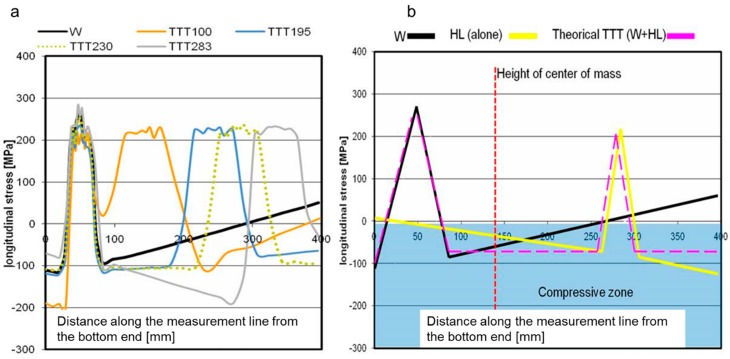
(**a**) Longitudinal residual stresses for different locations of the additional heat source (Figure 3a); (**b**) schematic superposition of residual stresses from FCAW welding and an additional heat source.

The residual stress values for different TTT processes (Figure 8a) show the slope of the residual stresses between the maximum peaks (a direct effect of the heat sources). The slope of these curves defines the direction of the final distortion. That final distortion is dependent on the intensity of the heat sources and the position of these sources with respect to the vertical center of mass. Figure 8b shows a simplified schematic of the theoretical residual stresses for the ideal htTTT process, helping to understand Figure 8a. Three possible slopes in the graphs are possible, and a horizontal curve is aimed for during the htTTT process.

**Figure 9:**
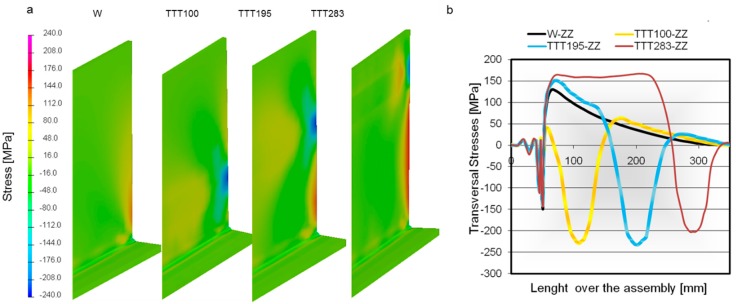
(**a**) Residual stresses for several htTTT tests; (**b**) stress comparison along the measurement line (Figure 3a).

The compressive stresses produced by htTTT determine the final distortion. Therefore, when the compressive stresses work below the center of mass, the structure becomes concave, while if the compressive stresses work over the center of mass, the final shape of the structure will be convex (Figure 9). The htTTT process makes it possible to find an optimal configuration of the propane torches that allows reaching the equilibrium of the compressive stresses, finally leading to zero out-of-plane distortion.

The study of the residual stresses helps us to understand the evolution of the distortion caused by the action of the htTTT. The stresses induced by the thermal loads and the edge effects contribute to producing the distortion of the assembly.

**Figure 10:**
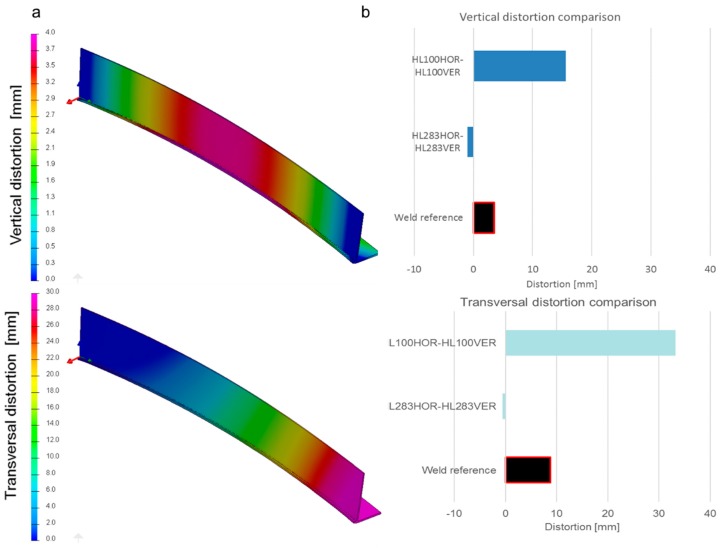
(**a**) P2 distortion results on the reference non-symmetrical weld (mm); (**b**) results of the distortion with htTTT.

Finally, the htTTT process was applied over non-symmetrical T-joints (P2) (Figure 10) and the same behavior was observed. For a non-symmetrical geometry, transversal distortion appears during a double-sided welding process. The vertical distortion of the T-joint reaches 3.52 mm while the transversal distortion reaches 8.74 mm (Figure 10b). Using htTTT at 690 °C on the web and wing, the values of the distortion change (Figure 10b).

When the auxiliary torches are located near the weld torches, the distortion increases as in the P1 case, while with an adequate positioning of the auxiliary htTTT torches, it is possible to reduce the distortion values. Therefore, the htTTT can be used to reduce the distortion during the welding process in both cases.

## 5. Discussion

htTTT has proven itself to be an efficient strategy to reduce distortions from welding processes. It causes the reduction in the distortion by adding new residual stresses, and therefore is a truly different process from the LSND (low stress no distortion) methodology, because the LSND aims to minimize residual stresses and the htTTT adds stresses into the assembly to minimize the distortion. htTTT was performed over two different T-joint geometries, and the results and conclusions obtained were similar. The temperature and position of the propane torches were studied, as both parameters are necessary to optimize the htTTT process for a given geometry of the component.

The htTTT does not affect the residual stresses on the bead zone if the auxiliary torches are far enough, and therefore the temperature over the bead remains almost constant, although future studies will be focused on their thermal behavior. If the temperatures are more uniform over the plates, the distortion at the final of the process is minimized. Moreover, other techniques such as preheating or slow cooling generate a temperature coupling that results in a more uniform distribution of temperature in the welded plates. The idea behind the htTTT is to minimize the weld distortion with compensative distortion applied in a different location.

## 6. Conclusions

In this work, htTTT was developed, studied and parametrized. The position and the heat power of the torches over a big symmetric T-joint were studied. Extreme reductions of the out-of-plane distortion in T-joints were obtained by applying htTTT. Therefore, high-temperature transient thermal tensioning can be used to fully eliminate the vertical distortion in T-joints.

The application of high-temperature transient thermal tensioning should be done far away from the bead and always over the neutral line. The higher power density source used for the TTT torches produces a distortion, and this phenomenon can be used to reduce the total distortion of the assembly. It has been found that the optimal htTTT configuration for minimizing the distortion of P1 T-joints involves applying the auxiliary propane torches at a distance of 330 mm from the flange with a 418 °C surface temperature. 

It has been shown that htTTT does not have any relevant impact on the residual stresses around the molten weld zone when the htTTT is performed far away from the focus of the weld. The improvement of the distortion behavior in the assembly is a result of the generation of new compressive stresses by the auxiliary torches. These new stresses produce compensation forces during the weld process, leading to a lower distortion at the end of the cooling. The process can be used with an asymmetrical setup of the stiffeners.

The use of higher temperatures (950 °C) results in higher distortion reductions, but they also cause metallurgical transformations, and therefore are not useful in the case of the material used in this study.
